# To Our Readers

**Published:** 1829

**Authors:** 


37. *To our readers.* The delay in the publication of this Number, has arisen from its running through those months which bring most occupation to medical men. The professional engagements of the Editor, have been such as to render it impracticable for him to prepare the matter for publication at an earlier period.

To the same cause must be ascribed the omission of the second essay on Medical Education. It will make the first article in the next Number, and appear on the first of January.

We have collected a variety of items of original intelligence, but want of time to condense and arrange them, has compelled us to postpone them to the third Number.

